# Diet Components, Immune Function and IgE-Mediated Food Allergy

**DOI:** 10.3390/nu17233669

**Published:** 2025-11-24

**Authors:** Rosina López-Fandiño

**Affiliations:** Instituto de Investigación en Ciencias de la Alimentación (CIAL, CSIC-UAM), Nicolás Cabrera 9, 28049 Madrid, Spain; rosina.lopez@csic.es

**Keywords:** allergy, aryl hydrocarbon receptor, diet, oral tolerance, retinoic acid, RORγt-expressing cells

## Abstract

Food allergies are rising globally, posing a multifactorial public health challenge driven by complex interactions among diet, immune development, and environmental exposures. This review highlights emerging insights into the cellular and molecular mechanisms by which specific dietary components, particularly vitamin A, fibre, indole compounds, and proteins, promote intestinal homeostasis. These nutrients act through both microbiota-dependent and -independent pathways, primarily in the small intestine, enhancing epithelial barrier integrity and supporting tolerogenic immune responses. Two key signalling axes, mediated by retinoic acid (RA) and aryl hydrocarbon receptor (AhR) ligands, converge to regulate RORγt-expressing immune cells, including group 3 innate lymphoid cells, TCRγδ^+^CD8αα^+^ intraepithelial lymphocytes (IELs), and Th17 cells, which are essential for secondary lymphoid organ development and barrier reinforcement. RA and AhR also guide the homing and specialization of diverse regulatory T cell subsets and CD4^+^ IELs, which collectively sustain peripheral tolerance to dietary antigens. Recent findings implicate RORγt^+^ antigen-presenting cells in the induction of peripheral Tregs during early life, particularly at weaning, underscoring a critical window for tolerance establishment. Microbial metabolites and commensal-derived signals further shape these immune pathways, reflecting the intricate interplay between host, diet, and microbiota in the regulation of oral tolerance.

## 1. Introduction

The global adoption of the Western diet, characterized by high intake of fat, protein, and ultra-processed foods but low fibre consumption, has paralleled the rise in non-communicable chronic diseases, which have now reached pandemic proportions [1]. Among them, the global prevalence of IgE-mediated food allergies has markedly increased to become a health burden worldwide and particularly in high-income, urbanized regions [2]. While genetic predisposition plays a role, this trend is attributed to a multifactorial interplay of early-life immune system development, dietary patterns, and environmental exposures [3,4].

The “hygiene hypothesis” proposed that reduced microbial exposure, due to improved sanitation and decreased contact with natural environments, impairs immune system maturation, triggering allergic disease [5]. The original hypothesis has progressively evolved into a more mechanistic framework, recognizing the cumulative influence of industrialization on the human microbiome while also identifying microbial signatures shared across distinct allergic manifestations [6]. Emerging evidence highlights the role of microbial diversity, environmental pollutants, and timing and route of allergen exposure in modulating allergic risk [7,8,9,10]. In general terms, it is assumed that increased food diversity in the first year is associated with reduced allergic sensitization and allergy outcomes in children [11,12]. This protective effect is thought to be mediated, at least in part, by the positive influence that enhanced exposure to dietary components exerts on gut microbial multiplicity and function [13,14]. The overall composition of the infant diet is an important factor in the development of allergic disease, with higher intake of fruits, vegetables, and home-prepared foods and, in general, higher healthy dietary scores in non-allergic children than in children who have a food allergy [15]. In contrast, Western dietary patterns are linked to reduced microbial diversity, potentially impairing mucosal immune tolerance and promoting proinflammatory responses to food antigens [16].

Diet is increasingly acknowledged as a modifiable factor in the prevention and management of allergic diseases. Many effects of dietary components are mediated by their impact on the intestinal microbiome [17]. There is evidence that diet shapes the intestinal microbiota of mice across diverse genotypes in a dose-dependent manner [18]. Similarly, in humans, diets exclusively composed of either animal- or plant-based products consistently modify the microbiome despite inter-individual variability [19]. Diet modulates microbial composition and can either promote eubiosis or contribute to dysbiosis, depending on both the pre-existing state of the microbiota and the nature, duration, and quality of dietary exposure [20]. Therefore, dietary responses remain highly personalized, highlighting the necessity for tailored nutritional interventions to modulate microbiome composition [21]. Multiple interactions exist between diet, microbiota, and immune responses that range from direct or indirect (metabolite-mediated) diet-induced changes in microbiota composition and activity to the generation of diet-derived microbial metabolites with immunomodulatory properties [22]. Furthermore, beyond its influence on microbial ecology, diet can directly modulate immune function, although the precise mechanisms through which specific nutrients regulate immune cell development and systemic immune responses remain incompletely understood [23].

The small intestine, the primary site of nutrient absorption, serves as a key location for oral sensitization to dietary proteins. In murine models of food allergy, the small intestine has been identified as a critical locus for the initiation and amplification of localized inflammatory responses following allergen exposure [24]. Compared to the colon, the microbiota of the small intestine is less well characterized. The continuous influx of digestive enzymes and bile, along with intermittent food delivery, shapes a microbial community with lower biomass and reduced diversity but highly adaptable to fluctuating environmental conditions and very susceptible to dietary influences [25].

This review explores the interplay between diet and immune responses, with a specific focus on the cellular and molecular mechanisms through which specific nutrients modulate immune responses and contribute to the establishment of oral tolerance. The analysis integrates findings from in vitro experiments, animal models, and, where available, epidemiological studies, acknowledging the limited availability of controlled human intervention trials. Particular attention is given to the impact of distinct dietary components on epithelial cell function and mucosal immunity in the small intestine due to their relevance in the pathophysiology and prevention of allergic disorders.

## 2. Vitamins

### 2.1. Vitamins D and E

Vitamin D insufficiency, prevalent worldwide, has been proposed to partly explain the increases in asthma and allergic diseases that we have witnessed during the past years [26]. Reduced maternal vitamin D levels during pregnancy, as well as vitamin D insufficiency in infancy, have been associated with an increased risk of developing food allergies, particularly during the second year of life [27]. Some studies have suggested that vitamin D, by signalling through the vitamin D receptor (VDR), a member of the nuclear receptor family of ligand-regulated transcription factors, can promote oral tolerance by favouring intestinal barrier function, inducting antimicrobial peptides, dampening IgE-mediated mast cell activation, driving a tolerogenic phenotype on dendritic cells (DCs), and acting directly on T cells to promote regulatory T (Treg) cells (supporting forkhead box P3 (Foxp3) and Cytotoxic T-lymphocyte associated protein 4 (CTLA-4) expression, as well as IL-10 and TGF-β production) [28,29,30,31]. Peripherally induced Treg cells play a critical role in establishing tolerance to exogenous antigens by controlling Th2 inflammation towards non-self-substances at mucosal surfaces [32]. These cells are typically characterized as CD4^+^ T cells expressing the IL-2 receptor α-chain (CD25) and the transcription Foxp3, which is widely regarded as a canonical marker of Treg cell identity, although, beyond Foxp3^+^ Treg cells, several distinct subsets of regulatory T cells have been identified, defined by the expression of additional lineage-determining transcription factors and by their secretion of immunosuppressive cytokines such as IL-10 and TGF-β [33]. However, the effect of vitamin D on Treg cells is complex and depends on the dose, environmental cellular milieu and on factors such as the age of the individual, pregnancy, and health or disease status, thus many questions remain regarding its role [34,35].

Maternal supplementation with α-tocopherol (α-T), a form of vitamin E, reduces development of allergic responses in offspring from allergic mice [36]. Similarly, early-life levels of α-T have been negatively associated with asthma development in children, particularly when levels of γ-tocopherol (γ-T) are low. Elevated γ-T concentrations seem to counteract the protective effects of α-T, suggesting that these two isoforms have opposing effects on the allergic response [37]. Results regarding food allergy are currently limited to animal models, showing that maternal supplementation with α-T reduces the incidence of food allergy and anaphylaxis in neonatal mice with skin barrier defects [38].

### 2.2. Retinoic Acid

Retinoic acid (RA), a vitamin A metabolite, plays a critical role in facilitating Foxp3^+^ Treg cell generation by acting to enhance signals derived from TGF-β, which is the limiting factor in this process [39,40]. Beyond augmenting TGF-β signalling, RA contributes to the stabilization of Treg cells by releasing IL-6 inhibition via the blockade of the expression of the IL-6 receptor [41,42]. Moreover, RA suppresses cytokine production by memory CD4^+^ T cells, particularly IL-4, and thus, it limits their capacity to antagonize TGF-β-mediated Treg conversion [42,43,44]. Notably, RA signalling also promotes the expression of *Rorc*, the gene encoding the transcription factor RAR-related orphan receptor gamma t (RORγt), thereby facilitating the differentiation of Foxp3^+^RORγt^+^ T reg cells. This subset constitutes the predominant population of colonic Treg cells in both mice and humans and plays a pivotal role in maintaining oral tolerance to commensal microbiota, as well as in regulating immune responses in the context of food allergy [45]. In the absence of TGF-β, in vitro, RA induces the differentiation of Foxp3^−^IL-10^+^ type 1 regulatory T (Tr1) cells, with immunosuppressive functions, even under inflammatory conditions [46]. Similarly, without TGF-β, RA induces the in vitro conversion of human group 2 innate lymphoid cells (ILC2s) into regulatory ILCs (ILCregs), which suppress CD4^+^ T cell and ILC2 proliferation via IL-10 production and express CTLA-4 and CD25, but not Foxp3 [47,48].

RA is produced through a two-step oxidation of vitamin A, catalyzed successively by widely expressed alcohol dehydrogenases and tightly regulated aldehyde dehydrogenases. Three different isoforms of the enzyme retinaldehyde dehydrogenase (RALDH), that oxidizes retinal into RA, have been described in mammal cells [49]. RA, due to its short half-life, must be synthesized locally, in close proximity to its target cells, and its concentration exhibits a decreasing gradient from proximal to distal regions of the intestine, which correlates with the RALDH activity of resident cells [50,51]. In mice, DCs from the lamina propria and intestinal lymphoid organs have for long been considered to be functionally specialized in the differentiation of Foxp3^+^ Treg cells largely due to their capacity to produce high levels of active TGF-β and express *Aldh1a2* (the gene encoding RALDH2) [39,40]. In addition to DCs, macrophages also express RALDH enzymes and contribute to a microenvironment conducive to mucosal tolerance [52,53].

**Figure 1:**
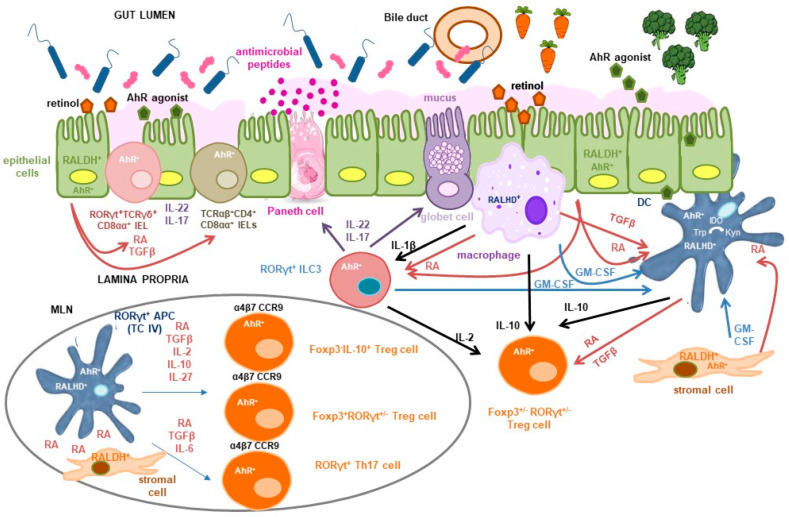
**Tolerogenic signals delivered by vitamin A and indole derivatives in the small intestine. Dietary retinol** absorbed from the intestinal lumen or secreted in the bile into the small intestine is metabolized into retinoic acid (RA) by constitutively retinaldehyde dehydrogenase (RALDH)-expressing cells, including intestinal epithelial cells (IECs) and lamina propria stromal cells. This serves as a primary source of RA that promotes its own synthesis by macrophages and dendritic cells (DCs). IL-1β, released by macrophages in response to microbial stimuli activates group 3 innate lymphoid cells (ILC3s) to produce granulocyte-macrophage colony-stimulating factor (GM-CSF), which together with GM-CSF produced by macrophages themselves and stromal cells, further enhances RALDH activity and induces RA and TGF-β secretion by DCs. RA directly activates RORγt^+^ cells, including ILC3s and TCRγδ^+^CD8αα^+^ intraepithelial lymphocytes (IELs), promoting secretion of IL-22 and IL-17. This cytokine cascade supports antimicrobial peptide and mucus production, thereby reinforcing epithelial barrier integrity. RA and TGF-β also contribute to the differentiation of tolerogenic TCRαβ^+^CD4^+^CD8αα^+^ double-positive IELs. Within intestinal lymphoid tissues such as mesenteric lymph nodes (MLN), RA facilitates regulatory T (Treg) cell induction by RORγt^+^ antigen-presenting cells (APCs) specific to microbial and dietary antigens. The relative concentrations of TGF-β, RA, IL-10, and IL-27 influence the differentiation of Foxp3^−^IL-10^+^, Foxp3^+^RORγt^−^, and Foxp3^+^RORγt^+^ Treg cell subsets. Additionally, RA, in concert with TGF-β and IL-6, promotes Th17 cell differentiation under homeostatic conditions, enabling IL-22 and IL-17 production to maintain epithelial integrity. RA modulates the pathogenic potential of Th17 cells by downregulating IL-6 and IL-23 receptor expression. It also induces intestinal homing receptors (α4β7 and CCR9) on activated T cells. Conventional DCs may deliver proliferative cues required for Treg cell maintenance, proliferation and function in the intestinal lamina propria. **Indole compounds**, derived from cruciferous vegetables or protein metabolism, activate the aryl hydrocarbon receptor (AhR), a ligand-dependent transcription factor expressed across IECs, stromal cells, and immune populations. In DCs, AhR activation suppresses proinflammatory cytokine production and enhances RA synthesis. It also upregulates indoleamine 2,3-dioxygenase (IDO), facilitating tryptophan (Trp) conversion to kynurenine (Kyn), an AhR agonist, and promotes TGF-β and IL-27 production, thereby influencing Treg cell differentiation. AhR signalling is critical for the development and function of RORγt^+^ cells, including ILC3s and Th17 cells, and supports the maintenance of natural IELs and the adaptation of induced IELs. As a transcriptional regulator of RORγt^+^ APCs, AhR may contribute to the induction of Foxp3^−^IL-10^+^, Foxp3^+^RORγt^−^, and Foxp3^+^RORγt^+^ Treg cells. It also modulates the balance between Treg and Th17 cells and regulates Treg cell intestinal homing and function. The co-expression of AhR and RA signalling pathways in multiple cell types suggests potential cross-regulation, with AhR influencing RA biosynthesis and RA modulating AhR activity, possibly through shared gene regulatory networks.

RA is both necessary and sufficient to induce *Aldh1a2* expression in DC precursors, thereby enhancing their autocrine production of RA and active TGF-β [50,51,54,55] (Figure 1). This RA-dependent positive feedback loop suggests that, in vivo, cells capable of constitutive RA production, independent of RA signalling, such as intestinal epithelial cells (IECs) and stromal cells from the lamina propria and mesenteric lymph nodes serve as an initial source to educate DCs [56,57]. IECs are a key source of RA, expressing high levels of *Aldh1a1* and converting dietary retinol absorbed from the intestinal lumen into RA. Retinol may also be metabolized into retinyl esters, stored in the liver, and later mobilized via the circulation or secreted in the bile into the small intestine [58] (Figure 1). RA and TGF-β produced by IECs imprint DCs with tolerogenic properties, promoting Treg cell induction both in murine models and in vitro or ex vivo human systems [59,60]. Furthermore, IECs secrete serum amyloid A (SAA) proteins, which are retinol binding proteins that transport retinol to intestinal DCs. DCs acquire retinol for conversion to RA through the LDL receptor-related protein 1 (LRP1) that mediates the uptake of SAA-retinol complexes [61].

Additional factors act synergistically to induce *Aldh1a2* expression and RALDH activity in intestinal DCs. Among these, granulocyte-macrophage colony-stimulating factor (GM-CSF; also known as colony-stimulating factor 2, *Csf2*) plays a key role. GM-CSF is produced by lamina propria macrophages, stromal cells and, notably, group 3 innate lymphoid cells (ILC3s) [54,57,62]. GM-CSF deficiency in mice also leads to impaired TGF-β production by DCs and reduced IL-10 secretion by macrophages, collectively compromising the differentiation of Foxp3^+^ Treg cells in both the small and large intestine [62]. GM-CSF production is regulated by signals from the commensal microbiota, which activate Toll-like receptors (TLRs) on intestinal macrophages, promoting IL-1β secretion. IL-1β, in turn, stimulates ILC3s via the IL-1β receptor pathway, driving GM-CSF production [62] (Figure 1). Additionally, microbiota-induced IL-1β signalling promotes IL-2 production by ILC3s, which contributes to the maintenance of Treg cells and the induction of oral tolerance to dietary antigens in the small intestine [63]. Beyond its indirect effects through GM-CSF, TLR signalling itself contributes directly to DC education by inducing *Aldh1a2* expression, as shown in murine DCs and in human DCs in vitro [50,55,64,65] (Figure 1). Interestingly, RA induces suppressor of cytokine signalling 3 (SOCS3) expression in DCs, which inhibits the TLR-driven production of proinflammatory cytokines that negatively affect Treg cell induction [66]. Furthermore, IL-4, which typically antagonizes Foxp3 expression, has been shown to synergize with RA to enhance the capacity of DCs to promote Foxp3^+^ Treg differentiation in the presence of TGF-β [67]. IL-4 and GM-CSF also act synergistically to strongly upregulate *Aldh1a2* expression and RALDH activity in murine bone marrow-derived DCs, effects that are further amplified by TLR stimulation and RA itself [54].

RA plays other roles in the establishment and maintenance of mucosal tolerance [68]. One of its key functions is the induction of gut-homing receptors, such as CCR9 and α4β7 integrin, on activated B and T cells, including Treg cells, thereby promoting their trafficking to the intestinal mucosa [69] (Figure 1). In B cells, RA synergizes with IL-6 to facilitate class-switch recombination to IgA, the principal antibody isotype secreted across mucosal surfaces. IgA modulates microbial colonization and has been proposed to inhibit allergic responses by preventing IgE binding to allergens [70], although other studies have questioned this protective role [71]. RA also impacts innate immunity by contributing to the development and transcriptional programming of conventional DCs in the bone marrow, and by enhancing their capacity to migrate to intestinal tissues [55,72,73]. Additionally, it facilitates the transmigration of mature proinflammatory DCs from the lamina propria to the intestinal epithelium where they acquire tolerogenic properties imprinted by both RA and mucus [74].

As mentioned above, RA signalling directly influences the differentiation and function of RORγt-expressing cells [75]. In addition to Foxp3^+^RORγt^+^ Treg cells, these include several other T cell subsets, such as natural T cell receptor (TCR)γδ^+^CD8αα^+^ intraepithelial lymphocytes (IELs), invariant natural killer T (iNKT) cells, and Th17 cells, as well as ILC3s, a population that comprises lymphoid tissue inducer (LTi) cells. These cell types cooperate to maintain intestinal barrier integrity primarily through the secretion of IL-22 and IL-17 [76]. RA influences intestinal immune responses by promoting IL-22 synthesis by ILC3s and TCRγδ^+^ CD8αα^+^IELs [77] (Figure 1). IL-22, constitutively produced in the small intestine and inducible in the colon under inflammatory conditions, acts specifically on IECs [78]. It promotes the production of antimicrobial peptides and mucus, facilitates epithelial repair, enhances symbiosis with commensal microbiota through IEC fucosylation, and aids in pathogen clearance [79]. Similarly, IL-17 enhances antimicrobial peptide production and contributes to barrier integrity and protection against pathogens [80]. Despite their protective roles, both IL-22 and IL-17 are implicated in the pathogenesis of various chronic inflammatory conditions, reflecting their context-dependent functions [76]. Interestingly, RA also induces the expression of IL-22 binding protein (IL-22BP), a soluble inhibitory receptor that neutralizes IL-22 activity, thereby serving as a regulatory mechanism to modulate IL-22-driven responses [81].

Recent murine studies have identified RORγt^+^ antigen-presenting cells (APCs), such as ILC3s [82], extrathymic autoimmune regulator (AIRE)-expressing cells or Janus cells [83], and Thetis cells (four different subsets with a hybrid phenotype between medullary thymic epithelial-like cells and DCs, with or without AIRE expression) [84] as key mediators in the induction of Foxp3^+^RORγt^+^ Treg cells that confer tolerance towards the microbiota. More recently, RORγt^+^ APCs have also been implicated in the induction of peripheral Treg cells specific to dietary antigens in mice, thereby contributing to oral tolerance to food. Notably, a distinct subset of Thetis cells, designated TC IV, has been shown to induce both Foxp3^+^RORγt^+^ and Foxp3^+^RORγt^−^ food antigen-specific Treg cells. This induction occurs prominently during the weaning period, a critical developmental window for Treg cell expansion and establishment of long-term immune tolerance, but also persists into adulthood, independent of microbiota composition [85,86,87]. Importantly, TC IV cells have also been identified in human tissues such as mesenteric lymph nodes, intestinal lamina propria and tonsils [86,88]. Despite some divergences in nomenclature and phenotype, other recent papers have also identified and described in mice the same or a closely similar tolerogenic APC type [89,90,91].

TC IV cells, predominantly enriched in lymph nodes draining the small and large intestine, are characterized by high levels of IL-2, MHCII, αvβ8 integrin, a potent activator of TGF-β, and RALDH2, which makes them responsive to RA, that enhances their tolerogenic function [85,89] (Figure 1). In contrast to their previously assumed roles, ILC3s and conventional DCs appear to be dispensable for establishing intestinal tolerance to dietary antigens across both early life and adult stages, prompting further investigation into their precise contributions to this process [85,86,87,90]. Nonetheless, DCs remain critical for maintaining other antigen-specific CD4^+^ T cell responses, indicating a functional specialization among APC subsets. Notably, migratory DCs that transport antigens from the lamina propria to gut-draining lymph nodes may provide proliferative signals essential for the maintenance of Treg cells, following their initial differentiation driven by RORγt^+^ APCs [85,89,92]. Furthermore, these DCs may contribute to Treg cell survival and functionality within the small intestinal lamina propria by delivering costimulatory cues within a tolerogenic microenvironment [93] (Figure 1).

## 3. Carbohydrates

### 3.1. Simple Sugars

Childhood consumption of high-fructose foods, sugar-sweetened beverages and high-carbohydrate ultra-processed products has been associated with an increased risk of allergic conditions, including food allergies [94]. A key factor implicated in this trend is the presence of advanced glycation end-products (AGEs), prevalent in Western diets and formed under high-sugar conditions. The consumption of AGEs has been positively associated with self-reported food allergy [95]. AGEs are hypothesized to trigger innate immune pathways that contribute to the initiation and amplification of allergic responses [96]. In vitro studies using human cell models have demonstrated that AGEs disrupt epithelial barrier integrity and elicit proinflammatory and Th2-skewed immune responses, pointing to a potential role in the observed rise in food allergy incidence [97]. In contrast to dietary sugar, that increases the inflammatory tone of the intestine [98], longer chain, non-digestible carbohydrates are highly beneficial to intestinal and immune health [99].

### 3.2. Fibre

Western diet is characterized by a low fibre intake, in most cases below the recommended daily range of 28–35 g for adults [100]. Fibre includes a broad array of carbohydrates that are not digestible by human enzymes or absorbable in the small intestine and provide an important substrate to the microbiota that inhabits the colon (in fact, it is its main energy source) to produce absorbable short-chain fatty acids (SCFAs). In mice only harbouring human-derived microbial communities, the transition from a low-fat, plant polysaccharide-rich diet to a high-fat, high-sugar Western diet causes significant alterations in microbiota structure and microbial metabolic pathways within a single day [101]. Diets low in fibre reduce the richness of the microbial community in the large intestine, causing dysbiosis and, in consequence, several diseases characterized by inflammation [102]. Notably, distinct dietary fibre structures elicit divergent and highly specific effects on microbiome composition, leading to shifts in the production of either propionate or butyrate or other bacteria-derived metabolites [103]. Thus, while dietary fibres are generally beneficial, certain components may trigger adverse immunological outcomes under specific conditions. For instance, it has been reported that inulin at a high concentration induces alterations in microbiota composition and metabolic activity that elevate bile acid levels (particularly cholic acid) and stimulates IL-33 production by stromal cells. This, in turn, activates IL-5-producing ILC2s, leading to excessive type 2 inflammation and allergy-prone responses in mice [104]. On the other hand, the immunological effects of dietary fibres are further modulated by the microbial fermentative capacity. In healthy individuals, a 17-week high-fibre dietary intervention revealed distinct immune response patterns linked to baseline microbial diversity [105]. Accordingly, individuals with low microbial gene richness show less improvement in inflammatory markers following fibre-rich dietary interventions [106]. Furthermore, in subjects lacking key fermentative microbes, certain fibres may have detrimental effects, due to unprocessed substrate accumulation or altered metabolite profiles that can exacerbate inflammation [107]. These findings underscore the complex, personalized nature of diet-microbiota-immune interactions and suggest that baseline microbiome composition may predict individual immunological responses to high-fibre diets.

A literature survey on epidemiological and intervention studies dealing with the consumption of dietary fibre for preventing or treating allergic diseases in humans did not identify any focused in food allergy. Nonetheless, studies in animal models have shown that fibre feeding confers protection from food allergy and increases the safety and effectiveness of oral immunotherapy protocols, mainly through the release of SCFAs by the colonic microbiota [108,109]. SCFAs, particularly butyrate, act through a combination of different immune and non-immune tolerogenic mechanisms to support intestinal homeostasis and prevent allergic sensitization [110]. Indeed, the microbiome of infants who later developed allergic sensitization is characterized by a reduced abundance of genes encoding enzymes involved in complex carbohydrate degradation and SCFA biosynthesis, particularly those responsible for butyrate production, impaired mucus integrity, heightened oxidative potential, and elevated levels of biogenic amines [111,112,113]. Interestingly, although absorbed SCFAs are largely metabolized in the liver and appear in only trace amounts in the peripheral circulation, they are found at exceptionally high concentrations in the portal venous system, particularly in small blood vessels draining the colonic mucosa. This localized enrichment suggests that SCFAs may influence the function of circulating immune cells as they transit through the portal circulation [114]. IECs and immune cells detect SCFA signals via G protein-coupled receptors (GPCRs) located on their surfaces or internalize SCFAs through monocarboxylate transporters. Inside the cells, SCFAs function as histone deacetylase (HDAC) inhibitors, inducing epigenetic changes that result in chromatin remodelling and the activation of diverse gene expression programmes, and promote metabolic changes that result in energy supply and cell activation [115].

Butyrate induces RALDH activity and increases RA conversion and active TGF-β production in human and mouse IEC lines and DCs [116,117,118]. As mentioned, this facilitates the differentiation of naive CD4^+^ T cells into colonic Foxp3^+^ Treg cells [119,120,121]. Furthermore, SCFAs directly modulate colonic Treg cells in mice, increasing their expression of *Foxp3* and *Il10*, proliferation and suppressive function [122,123]. In addition, through its conversion into acetyl-CoA, acetate induces the differentiation of immunosuppressive IL-10 producing B cells, in vivo and in vitro in both mice and humans, which effectively promote the differentiation of Treg cells [124]. Regarding the eliciting phase of food allergy, SCFAs suppress IgE-mediated activation of human and mouse mast cells in vitro and in vivo in the mouse, through a mechanism that involves GPR109A, prostaglandin 2 (PGE2), and epigenetic regulation (HDAC inhibition) [125,126].

A diet rich in saturated fats and simple carbohydrates, but depleted of dietary fibre, reduces growth rate and increases penetrability in the inner colonic mucus layer of mice, linked to loss of defined fibre-dependent bacteria that are beneficial for maintaining mucus function [127]. In particular, *Blautia coccoides* stimulates mucus growth through the production of propionate and acetate via activation of GPR43 [128]. Furthermore, dietary fibre deficiency compels the microbiota to utilize mucus glycoproteins as an alternative nutrient source, leading to the degradation of the colonic mucus barrier [129]. This fibre deficiency-driven expansion of mucin-degrading bacteria contributes to colonic barrier dysfunction and promotes a type 2 immune profile, potentially exacerbating food allergy susceptibility [130]. Indeed, in mice, mucin-2 induces tolerogenic factors in IECs and enhances IL-10, TGF-β, and RALDH2 expression in DCs, thereby supporting a regulatory immune environment [131]. SCFAs also modulate the proliferation and function of colonic ILC3s through activation of GPR43. This interaction promotes the production of IL-22, which in turn enhances the expression of mucus-associated proteins and antimicrobial peptides, thereby supporting the maintenance of epithelial barrier integrity and overall intestinal homeostasis [132]. Another pathway that contributes to intestinal homeostasis involves the ligand-activated transcription factor aryl hydrocarbon receptor (AhR), which is activated by indoles [133]. AhR plays a critical role in the development of ILC3s and Th17 cells, the production of IL-22, and the promotion of Treg cell homing and function [134]. Interestingly, the SCFA butyrate has also been shown to activate AhR in IECs, likely in synergy with its HDAC inhibitory activity, thereby further supporting intestinal health [135,136].

A low fibre diet also changes the composition of the microbiota at the small intestinal level, causing, in mice, a decrease in fibre fermenters. In adult mice, reintroduction of fibre in the diet restores the balance, although constant low-fibre diets cause defective vertical transmission of key microbes to offspring that can only be overcome by combining fibre supplementation with a fecal transplant [137]. Oral immunotherapy combining allergen with an inulin-based gel effectively restores the dysregulated ileal microbiome in sensitized mice, increasing the abundance of key metabolites such as guanosine and inducing long-lasting regulatory immune responses [138]. In particular, fibre contributes to the development of Segmented Filamentous Bacteria (SFB), a group of *Clostridium*-related organisms that attach to the ileal epithelium of their hosts [137]. SFB anchor to the surface of the small intestinal epithelium, without inducing an inflammatory response, and impact the generation of innate and acquired immunity. SFB colonization stimulates the production of antimicrobial factors and chemokines, induces the development of gut lymphoid tissue and increases fecal IgA concentrations in the lamina propria [139]. Notably, SFB enhance the number and effector activity of RORγt^+^ Th17 cells in the lamina propria. RORγt^+^Foxp3^+^ Treg cells are also induced by SFB in the small intestine, albeit at significantly lower frequencies compared to Th17 cells [121]. In addition to impair the differentiation of Th17 cells, low numbers of SFB also affect the development of TCRαβ^+^CD4^+^CD8αα^+^ IELs [137,140].

Colonization of the mouse small intestine with SFB as a single commensal microbe is sufficient to induce the differentiation of Th17 cells [141]. The TCR repertoire of intestinal Th17 cells has a minimal overlap with that of other intestinal CD4^+^ T cells, with most Th17 cells recognizing antigens encoded by SFB [142]. Th17 cells, characterized by the expression of the transcription factor RORγt and the production of IL-17 and IL-22, are specialized in the defence against extracellular pathogens, particularly at mucosal and epithelial barriers, although excessive activation leads to severe inflammatory and autoimmune disorders [143]. Although Th17 cells were originally characterized by their proinflammatory functions, accumulating evidence highlights the existence of non-pathogenic Th17 subsets that contribute to epithelial barrier maintenance and mucosal equilibrium. In fact, both IL-22 and IL-17 are involved in restraining the overgrowth of SFB and other bacterial species, revealing a balancing effect that helps to preserve intestinal homeostasis [143]. The functional dichotomy of Th17 cells, protective versus pathogenic, is largely context-dependent and influenced by the local microenvironment and the specific cues present during their differentiation [144,145]. RA plays a central role in this process by promoting the differentiation and stabilization of RORγt^+^Foxp3^+^ Treg cells while concurrently downregulating the expression of the IL-23 receptor, a key driver of pathogenic Th17 cell differentiation [121,146] (Figure 1).

A complementary function of SFB has to do with the development of tolerogenic TCRαβ^+^CD4^+^CD8αα^+^ T cells, referred to as double positive intraepithelial lymphocytes (DP IELs) or CD4^+^ IELs. These are induced IELs that derive from antigen-experienced CD4^+^ T cells, including Treg cells, that accumulate in the intestinal epithelium and reactivate the CD8 programme by acquisition of the CD8-lineage transcription factor Runx3 and loss of the CD4-lineage transcription factor T helper inducing POZ-Kruppel Factor (ThPOK) [147,148,149,150,151]. DP IELs display a regulatory function and collaborate with lamina propria Treg cells to favour tolerance to dietary antigens and protect against intestinal inflammation [150,152], albeit they may also exert cytotoxic activities depending on the context [147,153]. DP ILEs differentiate under the stimulus of the microbiota, with a diverse set of bacterial, dietary and environmental antigens and metabolites contributing to their optimal development [147,150,151,152]. TCR expression on CD4^+^ T cells and MHCII expression on IECs are required for the differentiation, but not maintenance, of conventional and Treg CD4^+^ T cells into DP IELs, suggesting that cognate antigen recognition is dispensable for their function [153]. Induction of an IEL phenotype also relies on intestinal signals like TGF-β and RA, which regulate CD103 expression for epithelial adhesion, and transcriptional cues including T-bet and IFN-γ or IL-27 signalling [148,149] (Figure 1).

The mechanism by which SFB induce DP IELs in the small intestine involves the production of IFN-γ, which drives the expression of MHCII on IECs and their antigen presentation ability, ultimately promoting the final maturation of CD4^+^ IELs intro double positive cells, although the source of IFN-γ may depend on the context [154]. According to Brabec et al. [155], IFN-γ can be produced by lamina propria Th1 cells, inducing the massive expression of MHCII on IECs along with a SFB-specific IEL response. This cognate DP IELs contribute to the renewal of the SFB-stressed gut epithelium by increasing its turnover rate, thereby complementing the Th17-mediated response aimed to control SFB colonization. On the other hand, Rodriguez-Marino et al. [140] suggested that group 1 innate lymphoid cells (ILC1s) and NKT cells produce IFN-γ in the absence of T cells under homeostatic conditions. In this way, SFB-induced MHCII expression enables IECs to present diverse microbial and dietary antigens, fostering a regulatory role for DP IELs.

SFB are widely present among vertebrates where they contribute to immune maturation, but it remains to be determined whether a human homologue of SFB exists with a function similar to that observed in mice. Although a human-specific SFB genome has been identified, its functional significance remains to be elucidated [156]. In humans, SFB constitute a minor fraction of the microbiota, with their peak abundance occurring between 21 and 36 months of age. This period coincides with increased expression of sIgA and Th17-associated markers, suggesting a potential role in postnatal immune development [157]. Regarding Th17 cell induction, human-derived *Bifidobacterium adolescentis*, along with other bifidobacterial species, has been shown to promote small intestinal Th17 cell differentiation. However, the mechanism of Th17 cell accumulation likely differs from that mediated by SFB [158]. *B. adolescentis* possesses polysaccharide transporters and degradation enzymes, but the relationship between dietary fibre and its immunomodulatory potential is yet to be fully established.

Dietary fibre may also exert microbiota-independent effects by modulating cells of the innate immune system or by protecting intestinal barrier function through actions on IEC maturation and tight junction proteins. These processes also depend strongly on the physicochemical properties of the fibre [159,160]. Microbiota-independent effects of fibre on innate cells rely on interactions with pattern recognition receptors (PRR) that bind carbohydrate structures and induce a variety of cellular responses, including production of cytokines and chemokines, which shape downstream adaptive immune responses. Key PRRs involved include *C*-type lectin receptors, such as dectin-1, dectin-2, and DC-SIGN [161], as well as TLRs [162,163]. Recognition of glycan structures from common allergens (for instance, peanut) by specific *C*-type lectin receptors can promote allergic responses. However, depending on the receptor involved, the physical nature of the carbohydrate ligand, and the strength of the engagement, activation of these receptors can also induce immune regulation and tolerance [164]. Similarly, TLR signalling plays dual roles, contributing to both allergy-associated and protective immune responses, influenced by genetic predisposition, environmental factors, and ligand characteristics [165].

In addition to their effects on immune cells, non-digestible polysaccharides have been shown to interact directly with surface receptors on IECs in in vitro cultures, such as TLRs, calcium-sensing receptors, and mannose receptors. These interactions activate a range of intracellular signalling pathways (including AMP-activated protein kinase (AMPK), mitogen-activated protein kinase (MAPK), and protein kinase (PK) pathways), which play key roles in the regulation of tight junction integrity and epithelial barrier function [166,167,168]. Dietary fibre also contributes to the strengthening of the epithelial barrier by modulating membrane composition, independently of changes in tight junction protein abundance, and by mitigating inflammation, further supporting barrier integrity and intestinal homeostasis [169,170]. Although in vivo studies remain limited, evidence supports the existence of rapid, microbiota-independent, beneficial effects of certain dietary fibres, such as psyllium, on intestinal barrier function. Psyllium exerts these effects in germ-free mice, albeit to a lesser extent than in specific pathogen-free counterparts, indicating that both microbiota-dependent and -independent mechanisms contribute to fibre-mediated barrier enhancement [171]. Similarly, fibre deprivation in germ-free mice has been shown to increase the expression of genes encoding the colonic type 2-inducing alarmins *Il25* and *Il33* [130]. A high rate of epithelial turnover and the subsequent increase in goblet cell numbers in the small intestine has been implicated in the effects of soluble fibres on baseline secretion of luminal sialylated mucins in rats [172]. However, other studies suggest that the proliferative effects of soluble fibre (specifically inulin at high concentrations) on colonic epithelial cells are strictly microbiota-dependent [173].

Oligosaccharides, key dynamic components of human milk, are well recognized for their beneficial effects on gastrointestinal mucosal barrier integrity, mucin production, and neonatal immune development, in addition to their established prebiotic properties [174]. While the majority of milk oligosaccharides transit undigested to the large intestine, approximately 1–3% are detectable in the blood and urine of breastfed infants, indicating partial absorption within the gastrointestinal tract at concentrations potentially sufficient to exert systemic immunomodulatory effects, although the underlying mechanisms remain incompletely understood [175]. The intestinal mucosal epithelium expresses high levels of galectins, a family of glycan-binding proteins with diverse extracellular and intracellular roles in regulating epithelial function as well as innate and adaptive immune responses [176]. Milk-derived lactose and oligosaccharides can inhibit the extracellular binding of galectins to their glycan ligands, thereby functioning as selective galectin inhibitors with therapeutic potential in a range of pathological conditions, or modulate galectin expression within the gastrointestinal epithelium [177,178]. Dietary intervention with oligosaccharides has been shown to suppress allergic effector responses in casein-sensitized mice [179]. The biological basis involves the secretion of galectin-9 by IECs, a soluble lectin that directly suppresses mast cell degranulation and promotes Th1 and Treg cell responses that counteract excessive type 2 immunity, preventing acute allergic symptoms in mice [180]. The mechanism by which galectin-9 fosters regulatory responses in vitro involves the induction of RALDH activity in DCs [181]. In humans, galectin-9 expression in small intestinal tissues positively correlates with serum levels of galectin-9 and IL-10, as well as with increased frequencies of peripheral Treg cells in non-allergic compared to food allergic individuals [182]. Furthermore, galectin-9 can restore the defective induction of *IL10* expression in naive peripheral B cells in food allergic patients, re-establishing the immune-suppressive function of Breg cells [183].

## 4. Phytochemicals

### 4.1. Indoles

Different plant-based foods contain ligands for the AhR, predominantly in the form of indole derivatives. Cruciferous vegetables, such as broccoli and Brussels sprouts, are particularly rich in indole-3-carbinol (I3C), which is converted in the acidic environment of the stomach into high-affinity AhR ligands [184]. AhR is broadly expressed in immune cells, IECs and stromal cells, and it plays a pivotal role in regulating mucosal immune responses.

As mentioned, AhR signalling is essential for the development and function of RORγt^+^ ILC3s and Th17 cells, as well as for the production of IL-22 [134] (Figure 1). Mice lacking AhR specifically in RORγt^+^ cells exhibit increased intestinal permeability to peanut allergens and heightened allergic responses, attributed to both IL-22-dependent and -independent contributions to epithelial barrier function [185]. However, barrier enhancement alone is insufficient to fully protect against food allergy, suggesting the involvement of additional immune-regulatory mechanisms [186]. Furthermore, analysis of a chromatin-accessible region within the *Rorc* locus, highly conserved between murine and human genomes, in the recently identified Treg cell inducers RORγt^+^ APCs, revealed a significant enrichment of AhR binding motifs. This finding implicates AhR as a key transcriptional regulator of RORγt^+^ APCs, also suggesting the potential involvement of TC IV cells in the pathogenesis associated with AhR-deficient mice [87]. Moreover, the data support a model in which both AhR and RORγt cooperatively regulate gene expression programmes that confer tolerogenic properties to these APCs [88]. In conventional DCs, where AhR expression is notably high, ligand-mediated activation promotes a tolerogenic phenotype characterized by enhanced RA synthesis and suppression of proinflammatory cytokines, thereby facilitating the differentiation of Foxp3^+^ Treg cells [187] (Figure 1).

AhR, along with signals such as TGF-β, TLR ligands, or interactions with Treg cells via CTLA-4, can induce DCs to express indoleamine 2,3-dioxygenase (IDO), which catalyzes the conversion of Trp into kynurenine (Kyn), which is itself an AhR agonist [188,189] (Figure 1). AhR signalling in naïve CD4^+^ T cells contributes to the differentiation of Treg cells capable of suppressing inflammation and autoimmunity [190]. AhR signalling also promotes the differentiation of IL-27-driven IL-10 producing Tr1 cells in vivo [191]. Moreover, AhR is expressed in peripherally induced Treg cells, where it influences gut homing and supports in vivo suppressive capacity [192,193] (Figure 1). Recent evidence also indicates that indole-containing phytochemicals stimulate IECs to express MHCII molecules via AhR signalling. These MHCII^+^ IECs interact with thymus-derived natural Treg cells, promoting their accumulation at the crypt tops of the colon [194]. Nutritional AhR ligands are also critical for IELs. Experiments in mice have shown that natural IELs rely on AhR signalling for tissue maintenance [195], while induced IELs use AhR pathways for local adaptation [196] (Figure 1). Consequently, diets lacking AhR ligands impair IEL maintenance, disturb microbial homeostasis, and heighten susceptibility to epithelial injury and immune activation [195].

Dietary supplementation with I3C alleviates allergic symptoms in peanut-sensitized mice, partially through the upregulation of *Aldh1* gene expression in the small intestine [197]. AhR activation has been shown to suppress food allergic responses in mice, primarily by modulating the frequency rather than the absolute number of Foxp3^+^ Treg cells [198]. Targeted deletion of *Ahr* in Treg cells eliminates the capacity of indole derivatives to induce RORγt^+^ Treg cell differentiation and confer protection against food allergy [199]. However, the pleiotropic effects of AhR ligands across multiple immune and epithelial cell types, combined with ligand-specific activity and variations in the route of administration, may account for inconsistencies between in vitro and in vivo findings and the variability observed across different experimental animal models [200].

### 4.2. Antioxidants: Isoflavones and Polyphenols

In human studies, increased oxidative stress prior to allergen exposure and inadequate antioxidant responses have been associated with a heightened susceptibility to occupational allergies [201]. At least in the case of inhalant allergens, an antioxidant-rich diet may contribute to allergy prevention [202]. In animal models, dietary isoflavones have been shown to suppress allergic sensitization and confer protection against peanut allergy [203,204]. Similarly, the anti-allergic potential of dietary polyphenols has been extensively investigated, with numerous studies documenting their anti-inflammatory, antioxidant, immunomodulatory, and microbiota-modifying effects [205]. Polyphenols can interact with food allergens through covalent and non-covalent binding, potentially altering protein conformation, promoting aggregation or crosslinking, and enhancing digestibility while reducing IgE-binding capacity. Such interactions have been proposed as a basis for developing hypoallergenic food products or novel immunotherapeutic approaches [206]. Experimental studies in murine models have shown that polyphenol administration during or after sensitization can reduce allergen-specific IgE levels, allergic symptoms, mast cell protease and histamine release, and Th2 cytokine production upon challenge, but the mechanism of action either at the cellular or molecular level is unclear [207,208,209,210,211,212]. Among the best supported properties of polyphenols is the inhibition of mast cell degranulation by acting on the high-affinity IgE receptor (FcεRI)-mediated pathway [213]. Depending on their structure, polyphenols modulate mast cell signal transduction via multiple mechanisms, including the downregulation of FcεRI surface expression, direct binding to FcεRI, and inhibition of key signalling molecules, such as Lyn or Syk kinases [214,215,216,217]. They also interfere with downstream signalling cascades involving MAPKs, protein kinase B (Akt), and Nuclear factor kappa-light-chain-enhancer of activated B cells (NF-κB), whose activation and nuclear translocation promotes the synthesis of inflammatory substances and reduce calcium influx by modulating calcium channel protein expression, a process critical for degranulation [218,219]. However, a more comprehensive understanding of the structural characteristics of polyphenols as they relate to absorption, metabolism, and bioavailability in humans is essential to fully assess their potential as antiallergic agents [220]. Researchers are beginning to decipher the specialized multi-enzyme system of specific members of the human gut microbiome, able to liberate aglycones from dietary phenolic glycosides with a broad diversity of effector functions, including the regulation of intestinal inflammation or, conversely, contributing to the increasing incidence of allergic diseases [221].

## 5. Proteins and Nucleic Acids

The essential amino acid L-tryptophan (Trp), an indole derivative, serves as a structural component of proteins and a precursor to a wide array of biologically active metabolites. Trp is metabolized primarily via four major pathways: the Kyn pathway, which accounts for approximately 95% of total Trp degradation, the serotonin pathway (also leading to melatonin synthesis), and the tryptamine and indole-pyruvate pathways [184]. The Kyn pathway is regulated at its rate-limiting step by IDO and tryptophan dioxygenase (TDO), and generates Kyn, a well-characterized agonist of AhR. As mentioned, activation of AhR modulates transcriptional programmes involved in multiple biological processes including immune responses. In parallel, bacterial metabolism of dietary Trp yields indole-based metabolites, another important class of AhR ligands. For instance, a Trp-rich diet promotes DP IEL development by upregulating Thpok via AhR activation in T cells, driven by indole derivatives produced by *Lactobacillus reuteri* [196]. Importantly, Trp metabolism is both shaped by the gut microbiota and, reciprocally, exerts significant effects on microbial composition and function through AhR-dependent and -independent pathways, in contrast with plant-derived I3C, which exhibits more limited microbiota-modulating capabilities [222,223,224]. Of note, high protein-diets reduce microbial diversity, increase microbial density and decrease intestinal barrier function, likely enhancing the antigenic load under conditions potentially leading to allergic reactions [171].

Experiments in mice have shown that, under physiological conditions, proteins are the principal drivers of peripherally induced Treg cell development in the small intestine, the primary site of nutrient absorption. These Treg cells are essential for establishing and maintaining the default state of oral tolerance, although their lifespan is relatively short compared to that of colonic Treg cells, which are predominantly induced by commensal microbial antigens, as they undergo rapid turnover upon withdrawal of dietary antigens [225]. Exposure of naïve, food antigen-specific CD4^+^ T cells to dietary peptides in homeostatic settings is thought to drive their differentiation into a heterogeneous population of hyporesponsive cells that lack canonical T helper lineage-defining markers and inflammatory effector functions, yet retain the potential to convert into Treg cells [226]. Dietary peptides in the duodenum trigger the cleavage of gasdermin D (GSDMD) in IECs, generating a non-pyroptotic fragment that enters the nucleus and promotes the transcriptional activation of class II transactivator (CIITA) and MHCII genes and the development of Tr1 cells, helping to maintain immune tolerance to food [227]. Furthermore, food-derived peptides contribute to epithelial barrier protection and, via TLR activation among other possible mechanisms, deliver tolerogenic signals to DCs. This stimulation induces RALDH enzyme activity, thereby promoting the generation of TGF-β-producing Foxp3^+^RORγt^+^ Treg cells [228,229,230]. In mice maintained on an antigen-free diet supplemented with amino acids, impairment in Treg cell generation and loss of oral tolerance is associated with profound transcriptional reprogramming of DCs in the small intestine [231]. These changes hinder key functional attributes of DCs, including their capacity for maturation, migration, activation, and induction of Treg cells in vitro. While protein deprivation is the primary driver of these alterations, shifts in the composition of the gut microbiota secondary to dietary changes also contribute to the modulation of DC function [231]. Lack of dietary antigens may also contribute to exacerbated type 2 responses in the small intestine through enhanced IL-25 production by tuft cells and subsequent ILC2 expansion [232].

In addition, compared with a diet devoid of whole protein, a protein-containing diet promotes clonal selection, maturation, epithelial adaptation, and cytotoxic programming of antigen-experienced conventional CD4^+^ T cells and Treg cells in the small intestine [24]. Increased diversity of dietary protein may have an additive influence. Notably, these effects occur independently of the microbiota, which, while not essential for DP IEL differentiation, serves to amplify the response through microbial stimulation [24]. The accumulation of DP IELs in the small intestine in response to dietary antigens strongly suggests their involvement in mediating tolerogenic responses to food [233]. Consistent with this tolerogenic function, the co-receptor CD8αα, expressed by DP IELs, has been shown to attenuate TCR antigen sensitivity [234], potentially restraining the cytotoxic effector mechanisms acquired during dietary protein-induced DP IEL differentiation. Furthermore, Lockhart et al. [24] demonstrated that during steady-state tolerance, DP IELs predominate; however, when this tolerance state is challenged with a Th2-driven inflammatory stimulus, there is a clonal expansion and accumulation of lamina propria Treg cells specific for dietary antigens, indicating a functional division of labour between these two T cell subsets.

Other diet-derived factors also contribute to the development and maintenance of natural IELs specifically in the small intestine. Notably, dietary nucleic acids activate innate receptors for RNA and DNA to induce IL-15 production by IECs. This cytokine response enhances the proliferation and survival of natural IELs independently of microbial signals [235]. Interestingly, nucleic acid supplementation promotes, through natural IELs (with the participation of both TCRγδ^+^CD8αα^+^ and TCRαβ^+^CD8αα^+^ subsets), oral tolerance in mouse models of food allergy by producing TGF-β1 that supports Treg cell induction by small intestinal DCs [235].

## 6. Lipids

Dietary fat increases intestinal permeability, promoting bacterial translocation, metabolic endotoxemia, and low-grade inflammation [236]. This compromised barrier facilitates allergen passage and disrupts antigen presentation, potentially impairing oral tolerance and enhancing food sensitization [237]. A high-fat, Western-style diet can trigger trained-immunity resulting in long-lasting enhanced innate immune responses to secondary inflammatory stimuli [238]. It also rapidly alters gut microbiota composition, gene expression and metabolic activity [101,239,240]. The type of fatty acids in a high-fat diet distinctly shapes the gut microbiota of mice: saturated fats (e.g., palm oil) have been reported to reduce beneficial Bacteroidetes, monounsaturated fats (e.g., olive oil) increase *Bacteroidaceae*, and omega-3 polyunsaturated fats (e.g., from flaxseed or fish oil) promote bifidogenic effects [241]. Nevertheless, though fat intake clearly impacts the microbiota, predictive bacterial markers for metabolic dysfunction remain undefined [242]. Among other consequences, reduced microbiome diversity induced by a high-fat diet suppresses MHCII expression in IECs via TLR2/ Myeloid differentiation primary response 88 (MyD88) and IFN-γ signalling pathways [243] that, as previously noted, may have implications for the development of DP IELs and Tr1 cells. Microbial alterations induced by a high fat diet in mice enhance susceptibility to food allergy [244]. Moreover, high-fat paternal diets alter microbiota and immune programming in offspring, increasing the risk of allergic sensitization via inherited dysbiosis, colonic inflammation, and reduced Treg cell frequencies [245,246]. In humans, maternal fat intake is linked to neonatal microbiome changes persisting until 4 to 6 weeks of age [247].

### n-3 Polyunsaturated Fatty Acids

Among dietary factors implicated in the rising prevalence of food allergy is the increased intake of *n*-6 polyunsaturated fatty acids (PUFAs) relative to *n*-3 PUFAs in Western diets during the latter half of the 20th century, primarily due to the replacement of animal fats with vegetable oils and margarines [248]. Linoleic acid (LA, 18:2*n*-6), abundant in corn, sunflower, and soybean oils, is the main *n*-6 PUFA, while α-linolenic acid (ALA, 18:3*n*-3), found in green plants, flaxseed, and walnuts, is the principal *n*-3 PUFA [249] (Russo, 2009). *n*-6 PUFA-derived mediators such as leukotrienes and prostanoids are proinflammatory and contribute to allergic disease [250,251]. However, *n*-3 PUFAs (mainly eicosapentaenoic acid, EPA, 20:5*n*-3, and docosahexanoic acid, DPA, 22:5*n*-3) compete as substrates with *n*-6 PUFAs, leading to less potent or anti-inflammatory mediators, like resolvins and protectins [252], and modulate the expression of proinflammatory genes [253].

Populations with diets high in marine *n*-3 PUFAs show lower incidence of inflammatory diseases [254]. Maternal fish intake and higher EPA levels in breast milk have been associated with reduced childhood allergy risk in observational studies, though the effects of fish intake during infancy and childhood are inconsistent [255]. On their part, meta-analyses of *n*3-PUFA supplementation during pregnancy and lactation or early life show mixed evidence regarding allergy prevention [256,257,258,259,260,261,262,263,264]. A recent study again highlights maternal *n*-3 supplementation during pregnancy and lactation as a potential strategy to reduce the risk of food allergies in offspring, particularly for egg and peanut sensitization, whereas childhood intake shows no comparable benefit [265].

*n*-3 PUFAs, EPA and DHA, inhibit LPS-induced maturation of DCs, reducing MHCII and co-stimulatory molecules (CD40, CD80, CD86), and thereby dampening T cell activation and proinflammatory cytokine production that supports Th1 responses [266,267]. These effects are partly mediated by suppression of the NF-κB pathway, a key regulator of inflammatory gene expression [252], with DHA being more effective than EPA in reducing NF-κB activation [268]. *n*-3 PUFAs also modulate adaptive immunity by altering T cell activation thresholds. In *n*-3 PUFA-enriched T cells, signalling through the TCR and CD28 is reduced, leading to impaired proliferation and cytokine secretion. However, these T cells can still respond under stronger stimulation [269]. This modulation is attributed to the incorporation of *n*-3 PUFAs into lipid rafts, ordered microdomains in the plasma membrane rich in cholesterol and sphingolipids, which disrupts raft organization and the recruitment and activation of signal-transducing proteins [270].

In vivo models of food allergy show that *n*-3 PUFA supplementation suppresses both Th1 and Th2 responses [271,272]. Fish oil supplementation increases tolerogenic CD11b^+^CD103^+^ DCs and intestinal Foxp3^+^ Treg cells, with DHA being more effective than EPA in preventing allergic responses and IgE/IgG1 production [272,273,274]. Offspring of mice fed diets rich in *n*-3 PUFAs exhibit altered gut microbiota, increased splenic IL-10 expression, and reduced allergen-specific IgE levels following sensitization, accompanied by attenuated allergic responses upon allergen challenge [246,275]. While fish oil has been reported to confer protective effects during both sensitization and effector phases of food allergy [276], other murine studies suggest that *n*-3 PUFAs suppress allergic symptoms independently of specific antibody levels by inhibiting mast cell activation [277,278]. DHA inhibits IgE class switching and production in human B cells through downregulation of CD40- and IL-4-mediated NF-κB and STAT6 signalling, without affecting IgA or IgG production [279]. Given that FcεRI signalling in mast cells depends on lipid raft integrity, *n*-3 PUFAs disrupt FcεRI localization within rafts, thereby interfering with degranulation, cytokine release, and the secretion of IL-4 and IL-13, ultimately reducing allergic symptom severity [280,281,282].

## 7. Conclusions and Future Directions

The rising incidence of food allergies represents a multifactorial challenge, driven by complex interactions among dietary habits, immunological development, and environmental exposures. A comprehensive understanding of how nutritional inputs together with environmental conditions, with or without microbial concurrence, shape immune tolerance is essential for devising effective prevention and intervention strategies. Specific dietary components, particularly vitamin A, fibre, indole compounds, and proteins, interact with the immune system through both microbiota-dependent and independent pathways to promote intestinal homeostasis. Two pivotal signalling pathways, mediated by RA and AhR ligands, converge to regulate the differentiation and function of RORγt-expressing immune cells, including ILC3s, TCRγδ^+^CD8αα^+^ IELs, and Th17 cells. These cell populations play indispensable roles in the development of secondary lymphoid structures and in fortifying the intestinal epithelial barrier through mechanisms such as tight junction maintenance, antimicrobial peptide secretion, and mucus production. RA and AhR ligands also critically influence the homing and functional specialization of different Treg cell subsets, including Foxp3^+^RORγt^+^, Foxp3^+^RORγt^−^, and Foxp3^−^IL-10^−^ Treg cells. These cells orchestrate active immune regulation essential for peripheral tolerance to dietary antigens. Additionally, RA and AhR signalling contribute to the development and tissue adaptation of CD4^+^ IELs, which collaborate with Treg cells to sustain intestinal immune equilibrium. Emerging evidence also implicates RORγt^+^ APCs in the induction of peripheral Treg cells during critical developmental windows, such as weaning. Diet-derived microbial metabolites and signals from the commensal microbiota further complemented these effects, modulating the immune function conductive to oral tolerance, reflecting the complexity of host–microbe–nutrient interactions.

Although many mechanistic pathways remain incompletely understood, current evidence suggests that overall dietary diversity may exert a more profound immunological impact than any single nutrient. This is likely due to the intricate interplay among multiple dietary constituents, host physiology, disease states, and microbial ecology in maintaining a tolerogenic mucosal environment. The concept of “precision nutrition”, tailoring dietary recommendations based on individual or population-level factors such as genetics, microbiome composition, dietary habits, and socioeconomic context, offers promising avenues for personalized allergy prevention and management. Future research should prioritize longitudinal cohort studies and targeted interventions that address microbiome restoration, optimal timing of allergen introduction, dietary modulation, and environmental mitigation. Such efforts will be critical in advancing our understanding of food allergy pathogenesis and in developing holistic, evidence-based strategies for reducing allergy burden across diverse populations.

## Data Availability

No new data were created or analyzed in this study. Data sharing is not applicable to this article.
